# Operationalizing a Free Flap Program for Head and Neck Reconstruction at a Veterans Affairs Hospital

**DOI:** 10.1002/oto2.80

**Published:** 2023-09-08

**Authors:** David J. Hernandez, William Xu, Yuli Lim, Jen L. Dong, Andrew T. Huang, Louisa Chiu, Samir Awad, Linda Joseph, Vlad C. Sandulache

**Affiliations:** ^1^ Bobby R. Alford Department of Otolaryngology–Head and Neck Surgery Houston Texas USA; ^2^ ENT Section, Operative Care Line Michael E. DeBakey Veterans Affairs Medical Center Houston Texas USA; ^3^ General Surgery Section, Operative Care Line Michael E. DeBakey Veterans Affairs Medical Center Houston Texas USA; ^4^ Anesthesia Section, Operative Care Line Michael E. DeBakey Veterans Affairs Medical Center Houston Texas USA

**Keywords:** 30‐day readmission, free flap, head and neck cancer, microvascular reconstruction, Veteran

## Abstract

**Objectives:**

We aimed to operationalize a head and neck microvascular free tissue transfer (MVFTT) program at a Veterans Affairs (VA) hospital with the emphasis on initiating radiotherapy within 6 weeks of surgery for cancer patients and minimizing readmissions.

**Study Design:**

Case series.

**Setting:**

Tertiary care VA hospital.

**Methods:**

A retrospective analysis was performed on consecutive head and neck MVFTT patients from May 1, 2017 and April 30, 2022. Demographics, patient and disease characteristics, per‐operative data and postoperative outcomes were recorded from the electronic medical record. We sought to compare our rate of 30‐day readmissions with those published in the literature.

**Results:**

One hundred and forty‐one procedures were performed in the queried timeframe. Eighty‐four percent (119) were performed after oncologic resections and 16% (22) were for nononcologic procedures. The rate of total flap loss was <1% and the rate of partial flap loss was 3.5%. For mucosal defects, the fistula rate was 2.3%. The rate of return to the OR for any reason within 30 days was 7.8%. The 30‐day readmission rate was 6.4% while the rates reported in the literature range from 13% to 20%. One hundred and four patients required postoperative radiotherapy (PORT) and 76% started PORT within 42 days of surgery.

**Conclusion:**

Operationalizing a head and neck MVFTT program with a VA hospital is safe and allows for the successful delivery of multimodality treatment to cancer patients. These resources can be expanded for the care of head and neck cancer treatment sequelae, such as osteoradionecrosis, and other nononcologic patient needs.

Microvascular free tissue transfer (MVFTT) has been established as the gold standard for reconstruction after major head and neck oncologic resection.^1^ The adoption of free flap reconstruction into oncologic programs has facilitated the use of multimodality treatment, namely surgery and adjuvant radiation, by allowing for expedited healing prior to initiation of external beam radiation therapy (EBRT).^2^ This has been particularly beneficial for the treatment of head and neck squamous cell carcinoma (SCC) of the oral cavity and larynx/hypopharynx and to a lesser degree to select populations of patients with oropharyngeal cancer. The epidemic rise of HPV‐mediated oropharyngeal SCC (OPSCC) has given way to the use of primary radiation, minimally invasive/transoral surgery, and occasionally de‐escalation protocols for low‐risk disease in nonsmokers.^3^, ^4^ However, there remains a significant subset of patients, particularly Veterans, with intermediate (p16+ smokers) and high‐risk (p16−) OPSCC that requires multimodality treatment which often involves complex resection and MVFTT.^5^, ^6^, ^7^, ^8^, ^9^, ^10^, ^11^, ^12^, ^13^, ^14^

Veterans present with a disproportionate burden of head and neck cancer in several respects. Across all disease sites, they present with more advanced T‐ and N‐classification. As a result, Veterans with oral cavity and laryngeal SCC often necessitate a combination of surgery and adjuvant EBRT which makes it essential to minimize postoperative wound healing complications, particularly fistula formation. Within the oropharynx site, Veterans present with a greater burden of high‐ and intermediate‐risk disease which also increases the need for a combined surgery + EBRT approach.^2^, ^10^, ^11^, ^12^, ^15^, ^16^

We sought to operationalize a head and neck MVFTT program within a tertiary Veterans Affairs (VA) institution without the need for outsourcing the surgical expertise to an outside institution in order to avoid fragmentation of the oncologic care of the patients, which has been shown to reduce oncologic success.^17^, ^18^, ^19^ As we built the MVFTT program, we set two primary goals. The first was to prioritize adjuvant treatment initiation in a timely fashion in compliance with National Comprehensive Cancer Network (NCCN)—and now, American College of Surgeons/Commission on Cancer (ACS/CoC)—guidelines. From a reconstructive standpoint this meant using MVFTT as a tool to maximize a patient's ability to heal after oncologic resection. The second was to prioritize patient and caregiver independence upon discharge over a reduction in length of stay (LOS). With these goals in place for the oncologic patients, we implemented a head and neck MVFTT program at our institution that addressed oncologic and nononcologic reconstructive needs. We hypothesized that with the above goals, we would achieve a low rate of 30‐day readmissions for patients compared to the national average of 19.4% (Goel).

## Materials and Methods

We retrospectively analyzed data from an institutional cohort of patients that underwent MVFTT for defects of the head and neck region. Following approval from Baylor College of Medicine and Michael E. DeBakey Veterans Affairs Medical Center (MEDVAMC) Institutional Review Boards, we reviewed the records of patients who met inclusion criteria. Inclusion criteria included: (1) MVFTT of a mucosal or soft tissue defect of the head and neck region and (2) surgery performed between May 1, 2017 and April 30, 2022. Patient demographics, procedure details, tumor and treatment characteristics, comorbidities as measured by the Head and Neck–Charlson Comorbidity Index (HN‐CCI)^20^, perioperative complications, and clinical outcomes were retrospectively obtained from the existing electronic medical record.

As part of our MVFTT protocol, all patients were transferred to the surgical intensive care unit (SICU) postoperatively for hourly nursing flap checks for the first 72 hours. Thereafter, patients were transferred to an intermediate care unit (IMU) with every 2 hours nursing flap checks. By postoperative day (POD) 5, flap checks were reduced to every 4 hours, which allowed all patients to be transferred to regular floor status. On POD 1, attempts were made to mobilize the patient to a chair, or ambulate with assistance, and the Foley catheter and arterial line were removed, with rare exceptions. Antibiotics were routinely continued for 1 week postoperatively, with ampicillin‐sulbactam as the preferred option. Physical Therapy (PT) and Occupational Therapy (OT) services were consulted on POD 1 for continued progress with mobilization. Drains were removed when their output reached less than 30 mL for 24 hours. Patients were decannulated prior to discharge when appropriate. Select cancer patients underwent EBRT simulation prior to discharge and 100% of remaining cancer patients slated for adjuvant EBRT received a simulation date prior to discharge (EBRT operations are co‐located with surgical and inpatient care operations at our facility). Patients were discharged when they and, if applicable, their caretakers/family were able to demonstrate independence with wound care, tracheostomy, and gastrostomy tube care, if applicable. Many patients, particularly those patients who required adjuvant EBRT, were able to take advantage of the institutional Community Living Center (CLC) in the postoperative setting, which is a skilled nursing unit located on‐campus. We consistently avoided discharging patients to non‐VA skilled nursing facilities (SNFs) or long‐term acute care facilities (LTACs) due to the challenges with transportation and coordination of adjuvant EBRT with these options.

## Results

During the study period our team performed 141 procedures. Of these procedures, 119 were performed for cancer indications (Table 1A) and 22 were performed for noncancer indications (Table 1B). The vast majority of patients were male and more than 65% in both categories were between 61 and 75 years of age. Mean tobacco exposure in the cancer group was 34.6 pack years and in the noncancer group it was 24.1 pack years. Head and Neck–Charlson Comorbidity Index (HN‐CCI) was 0.29 in the cancer group and 0.41 in the noncancer group. Over 20% of cancer patients and 68% of noncancer patients had a history of prior radiation. In the cancer cohort, 63% of procedures were performed for a mucosal defect. In the noncancer cohort, 73% of procedures were performed for a mucosal defect. The most common primary cancer sites were the oral cavity and the skin.

**Table 1A:** Patient and Disease Characteristics (Cancer Patients)

Variable	Value	Percent
Total	119	
Age		
<60	19	16
61‐75	86	72.3
>75	14	11.8
Mean	68	
Median	70	
SD	7.9	
Sex		
Male	117	98.3
Female	2	1.7
Pack years		
mean	34.6	
median	35	
range	0‐140	
SD	32.4	
HN‐CCI		
Mean	0.29	
SD	0.54	
prior EBRT		
Yes	27	22.7
No	92	77.3
Prior chemo		
Yes	15	12.6
No	104	87.4
Pathology		
SCC	97	81.5
BCC	11	9.2
Melanoma, cutaneous	3	2.5
Sarcoma	4	3.4
Adenocarcinoma	1	0.8
Adenoid cystic carcinoma	1	0.8
Polymorphous adenocarcinoma	1	0.8
Poorly differentiated carcinoma	1	0.8
Aggressive fibrous osteoma	1	0.8
Defect		
Mucosal	75	63
Skin/soft tissue	44	37
Primary disease site		
OC	35	29.4
OP	17	14.3
Larynx/hypopharynx	16	13.4
Cutaneous	38	31.9
Sinonasal	6	5.0
Lacrimal gland	2	1.7
Parotid	1	0.8
Neck	4	3.4
T stage		
is	1	0.9
0	3	2.8
1	11	10.3
2	30	28.0
3	31	29.0
4	31	29.0
N stage		
0	56	52.3
1	12	11.2
2	18	16.8
3	21	19.6

Abbreviations: BCC, basal cell carcinoma; EBRT, external beam radiation therapy; HN‐CCI, Head and Neck–Charlson Comorbidity Index; is, in situ; OC, oral cavity; OP, oropharynx; SCC, squamous cell carcinoma; SD, standard deviation.

**Table 1B:** Patient and Disease Characteristics (Noncancer Patients)

Variable	Value	Percent
Total	22	
Age		
<60	5	22.7
61‐75	15	68.2
>75	2	9.1
Mean	66	
Median	70	
SD	10	
Sex		
Male	22	100
Female	0	0
Pack years		
Mean	24.1	
Median	10	
Range	0‐129	
SD	31.9	
HN‐CCI		
Mean	0.41	
SD	0.67	
Prior xrt		
Yes	15	68.2
No	7	31.8
Prior chemo		
Yes	9	40.9
No	13	59.1
Indication for flap		
ORN	10	45.5
ONJ	1	4.5
Bening tumor	2	9.1
Facial paralysis	1	4.5
Chronic wound	4	18.2
Trauma	1	4.5
Necrotizing fasciitis	1	4.5
Dysfunctional larynx	1	4.5
Pharyngeal stenosis	1	4.5
Defect		
Mucosal	16	72.7
Skin/soft tissue	6	27.3

Abbreviations: ONJ, osteonecrosis of the jaw; ORN, osteoradionecrosis; SD, standard deviation.

The most common flap was the anterolateral thigh (ALT) flap at 62.4%, followed by the forearm flap at 19.9% (Table 2). Mean surgery time across the study period was 9.6 hours (Table 3). Nearly all (91%) procedures required intermittent pressor support, and 21% of cases were performed in the presence of a continuous pressor drip for at least a portion of the case. Blood transfusions were performed in 35% of cases and 69% of patients underwent intraoperative tracheostomy. Mean LOS for the entire cohort was 13.2 days; 6.4% of patients underwent readmission (all reasons) within 30 days of the primary surgery and 2.8% of patients died within 90 days of the primary surgery. Among 9 30‐day readmissions, 5 (56%) were related to a wound, infection, or fistula, 3 (33%) were for failure to thrive/dehydration, and 1 (11%) was due to chyle leak.

**Table 2:** MVFTT Selection

Flap selection	Number	%
ALT	88	62.4
Forearm, all	28	19.9
Forearm, radial	18	12.8
Forearm, ulnar	10	7.1
Fibula	15	10.6
Scapula	9	6.4
Gracilis	1	0.7

Abbreviations: ALT, anterolateral thigh; MVFTT, microvascular free tissue transfer.

**Table 3:** Operative and Perioperative Statistics

	All	ALT	Forearm, all	Fibula	Scapula	Gracilis
Total (*n*)	141	88	28	15	9	1
Intra‐op pressor, intermittent (%)	91	92	86	100	78	100
Intra‐op pressor, continuous drip (%)	21	20	18	7	11	0
Intra‐op blood transfusion (%)	35	37	11	40	56	0
Tracheostomy (%)	69	58	33	73	78	0
Length of stay, mean (days)	13.2	12.8	11.6	15.6	17.9	13
Medical complication (%)	26	23	25	33	56	0
30 day readmission (%)	6.4	6.8	7.1	0	11	0
Death within 90 days	2.8	4.5	0	13	0	0
Takeback within 30 days, any reason (%)	7.8	11.4	0	0	11	0
Flap loss, total (%)	0.7	0	3.6	0	0	0
Flap loss, partial (%)	3.5	5.7	0	0	0	0
Fistula for mucosal flaps (n = 88; %)	2.3	4.1	0	0	0	n/a

Abbreviation: ALT, anterolateral thigh.

Total flap loss rate was <1.0% and the fistula rate was 2.3% for flaps performed for mucosal defects. Return to OR within 30 days of the primary surgery included: 1 donor site skin graft placement, 1 hematoma evacuation, 2 regional flaps required due to partial flap loss, 2 skin grafts for the recipient site, 2 regional flaps for a fistula formation, and 3 revisions of the microvascular anastomosis. All reoperations occurred prior to discharge for patients discharged to the CLC. Thirty‐seven (27%) of veterans were discharged to the CLC (on‐campus skilled nursing unit), 101 (73%) were discharged home, and only 1 (<1%) was discharged to a non‐VA facility.

**Figure 1:**
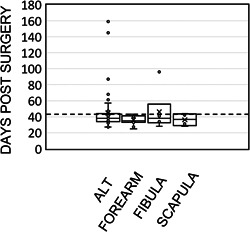
PORT characteristic. Individual patient‐level data for each major MVFTT procedure type indicating days between surgery and PORT initiation for those cancer patients slated for PORT. MVFTT, microvascular free tissue transfer; PORT, postoperative radiotherapy.

Among the patients who underwent reconstruction for a cancer indication, 104 (92%) underwent postoperative radiation therapy (PORT) (Table 4); of these patients, 76% started PORT within the recommended 42 days postsurgery. Of note, this included 12 patients which underwent reirradiation following the reconstruction due to a previous history of head and neck radiation. Mean time to radiation was 41.2 days (median = 36 days) (Figure 1). Thirty (34%) patients that received PORT (total n = 86) were initially discharged to the CLC, and 56 (65%) of these patients that received PORT were discharged home. One patient (1%) receiving PORT was discharged to a non‐VA facility. There were no statistical differences in timely PORT delivery based on patient discharge to the CLC versus home (80% vs 73%, *P* = .46).

**Table 4:** PORT Initiation Statistics

Flap type	PORT (n)	Timely PORT (%)	Time to PORT (days), mean	Standard deviation	Time to PORT (days), median
ALT	55	69	44.6	23.3	38
Forearm, all	19	95	35.9	5.3	35
Forearm, radial	11	100	35.8	4.9	35
Forearm, ulnar	8	88	36	5.9	37.5
Fibula	6	83	46	22.8	38
Scapula	4	75	36.3	6.9	36.5
All	84	76	41.2	18.7	36

Abbreviation: PORT, postoperative radiotherapy.

**Figure 2:**
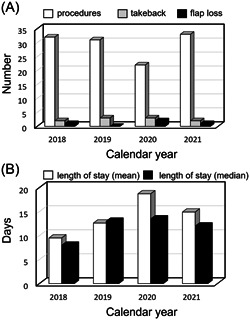
MVFTT delivery over time. (A) Number of procedures performed per year compared to number of OR takebacks and partial or total flap loss. (B) Length of stay (mean and median) over time in days. MVFTT, microvascular free tissue transfer.

Throughout the study period, we experienced a stable volume of total procedures with consistent takeback and flap loss rates (Figure 2A) apart from 2020, during which the total procedure number decreased commensurate with the COVID‐19 pandemic. In the same year, we experienced a concomitant rise in LOS secondary to difficulties with postoperative placement and the need for extended in‐house oncologic interventions (eg, postoperative simulation) which increased secondary to difficulties with access to the institution (Figure 2B).

## Discussion

The availability of MVFTT for complex head and neck oncologic defects has played a vital role in the timely delivery of multimodality oncologic treatment, namely adjuvant EBRT following oncologic surgery. Only recently, the ACS/CoC introduced the first quality metric for head and neck squamous cell carcinoma: initiation of adjuvant EBRT within 6 weeks of surgery.^21^ This metric, along with the long‐standing NCCN guideline, is driven by data demonstrating improved oncologic outcomes with compliance within this timeline.^17^, ^22^, ^23^, ^24^ Thus, the primary objective of a reconstructive endeavor in this setting is to provide the patient with a safe reconstruction, or wound, to allow for the safe delivery of PORT in the prescribed time. This has been the priority over the years at our institution,^25^, ^26^ and it has resulted in 76% compliance with this metric in the present cohort, which includes 11 patients that received re‐irradiation. A prior study by our group comparing oral cavity to oropharyngeal squamous cell carcinoma patient outcomes identified the advantage of tailoring the reconstruction to focusing on the patient healing expeditiously in order to initiate adjuvant treatment on time.^2^ This approach, along with the horizontal and vertically integrated structure of the VHA, plays a vital role in our ability to achieve rates of timely PORT that compare favorably with leading cancer centers in the United States.^27^

Our 30‐day readmission rate (6.4%) was lower than those reported by a large Nationwide Readmissions Database study that included 9487 patients (19.4%).^28^ Further, our readmission rate was half the rate reported by Graboyes et al (13%) in a cohort study of 493 patients undergoing head and neck microvascular free flap reconstruction.^29^ This is an important metric due to the potential impact on timely delivery of PORT and the cost associated with the additional hospitalization. Goel et al reported a mean cost per readmission of $15,916.^28^ The low readmission rate in our cohort is in line with our emphasis on patient and caregiver independence with respect to the ability to care for the patient's wound(s), tracheostomy, and gastrostomy tubes, if present, prior to discharge. Thus, we de‐emphasized the focus on reducing LOS to ensure that we minimized drains in place at time of discharge and allowed for safe decannulation where appropriate prior to discharge. Despite access to our on‐campus SNF (CLC) for 37 (27%) of the patients, LOS remained elevated due to logistic and bed availability restrictions for patients being considered for admission to the CLC. Furthermore, the pandemic introduced additional testing and occasional quarantine requirements prior to admission to this unit. Twenty‐one (43%) of the 49 patients that were managed perioperatively with a tracheostomy were decannulated prior to discharge. Despite the lack of attention to reducing LOS, our cohort LOS was only slightly higher than that of the NSQIP database cohort reported by Cannady et al (13.2 vs 11.6 days).^30^ Ultimately, our hypothesis was correct that our 30‐day readmission rate would be lower than reported national standards with the approach we implemented.

Our complication rates were relatively low when compared to national rates. The rate of return to the OR within 30 days was 7.8%. A NSQIP study of head and neck cancer‐free flap reconstruction included 1643 flaps and found a 19.7% rate of return to the OR within 30 days.^30^ Of the 11 patients in our cohort that returned to the OR within 30 days, this included 1 patient that underwent a donor site skin graft placement and 2 patients that only underwent skin grafting at the recipient site. Antibiotic use at our institution is not consistent with suggested efficacy of shorter durations for similar reconstructive head and neck procedures;^31^, ^32^, ^33^ however, our institutionally low rate of fistula formation (particularly after laryngectomy and flap reconstruction)^34^ attributed to multilayer reconstructive technique is achieved in the setting of the current more liberal use of antibiotics, typically 1 week postoperatively.

A VASQIP study published in 2018 demonstrated that for head and neck surgeries performed over time, the rate of complications has decreased for most procedures with the exception of total laryngectomy and free flap surgery, which saw a significant increase in complication rates from 1995‐2000 to 2011‐2015.^35^ However, the authors note that this may be due to the expanded application of these surgeries to older patients with greater comorbidity burden. Nonetheless, this study highlights the challenges and risks with performing head and neck free flap surgery in a Veteran patient population.

The availability of an on‐campus CLC unit was a great asset whenever patients were discharged to this unit, because it allowed for weekly rounding to ensure patient comfort and safety as they made the transition to their adjuvant treatment. During the height of the COVID‐19 pandemic in 2020, patient access to the CLC unit was more restricted and often delayed due to newly implemented protocols and restrictions for admission. These changes led to longer inpatient LOS in 2020 and into 2021 (Figure 2B).

While the treatment of oncologic patients was the impetus for operationalization of our MVFTT program at the MEDVAMC, the utilization of MVFTT for nononcologic indications is a direct benefit to the institution and its patients, particularly those patients with treatment sequelae complications such as osteoradionecrosis (n = 10), chronic wounds (n = 4), dysfunctional larynx (n = 1) and pharyngeal stricture (n = 1). Arguably, many of these patients are more challenging to treat from a reconstructive standpoint due to the significantly higher rate of prior head and neck radiation in the noncancer cohort (68% vs 23% in the cancer cohort, *P* < .01). Other indications for MVFTT in this series included osteonecrosis of the jaw (medication‐induced), facial paralysis, trauma (gunshot wound), necrotizing fasciitis wound, and benign tumors. Overall, flap outcomes were quite good. The takeback rate (all returns to OR within 30 days) was 7.8%. We only encountered 1 total flap loss (0.7%) which occurred in 2017. Our partial flap failure rate was 3.5%. Thus, depending on the measurement used for flap success (total loss or total and partial loss), our rate of success was 99.3% or 95%, respectively. Furthermore, the last takeback for anastomotic revision was in October of 2020.

There is a paucity of literature on free flap outcomes in the Veteran patient population. Myers reported comparable flap outcomes for patients across 3 different patient populations—private, public/county, and VA.^36^ Another study by Myers et al^37^ demonstrated a success rate of 93% after performing 55 flaps at a VA institution compared to the 95% success rate (based on no partial or total loss) in our 141 patients. Myers' reported LOS was 16 days (13.2 for our cohort). Our study, which is the largest study of head and neck free flap outcomes at a VA institution, indicates it is feasible to perform head and neck MVFTT in a tertiary VA institution with good surgical and oncologic outcomes. Costs associated with head and neck MVFTT have been estimated at $47,681 (SE $5481) per patient.^38^ Without considering additional costs derived from higher rates of flap failure, readmission, and treatment fragmentation, the total cost of MVFTT for our 141 patient cohort would have exceeded $6,700,000 if performed through the Community Care program.

## Conclusion

In a VA institution that is vertically and horizontally integrated, operationalizing a head and neck MVFTT program allows for optimal delivery of timely multimodality head and neck cancer treatment in a Veteran patient population. We demonstrate that a program is both feasible and safe with clinical outcomes comparable, if not better than, national standards. This then translates into the availability of resources and expertise to treat a multitude of other head and neck defects for non‐oncologic pathologies. Our study is the largest single institution head and neck free flap Veteran cohort in the literature, to our knowledge, and may serve as a template for other VA programs around the country.

## Author Contributions

**David J. Hernandez**, MD, concept, acquisition of data, interpretation of data, drafted manuscript, revised manuscript, final approval; **William Xu**, BS, acquisition of data, interpretation of data, revised manuscript, final approval; **Yuli Lim**, BA, acquisition of data, interpretation of data, revised manuscript, final approval; **Jen L. Dong**, BS, acquisition of data, interpretation of data, revised manuscript, final approval; **Andrew T. Huang**, MD, concept, interpretation of data, revised manuscript, final approval; **Louisa Chiu**, MD, concept, interpretation of data, revised manuscript, final approval; **Samir Awad**, MD, concept, interpretation of data, revised manuscript, final approval; **Linda Joseph**, MD, concept, acquisition of data, interpretation of data, revised manuscript, final approval; **Vlad C. Sandulache**, MD PhD, concept, acquisition of data, interpretation of data, drafted manuscript, revised manuscript, final approval.

## Disclosures

### Competing interests

Contents do not represent the views of the US Department of Veterans Affairs or the US Government. The authors report no conflicts of interest for the existing work.

### Funding source

This material is the result of work supported with resources and the use of facilities of the Michael E. DeBakey VA Medical Center. V. C. S. is supported by a Career Development Award from the Veterans Administration Clinical Science Research and Development division (1IK2CX001953).
